# Extracellular nucleotides as novel, underappreciated pro-metastatic factors that stimulate purinergic signaling in human lung cancer cells

**DOI:** 10.1186/s12943-015-0469-z

**Published:** 2015-11-24

**Authors:** Gabriela Schneider, Talita Glaser, Claudiana Lameu, Ahmed Abdelbaset-Ismail, Zachariah Payne Sellers, Marcin Moniuszko, Henning Ulrich, Mariusz Z. Ratajczak

**Affiliations:** Stem Cell Institute at James Graham Brown Cancer Center, University of Louisville, 500 South Floyd Street, Louisville, KY 40202 USA; Departamento de Bioquímica, Instituto de Química, Universidade de São Paulo, São Paulo, Brazil; Department of Regenerative Medicine and Immune Regulation, Medical University of Bialystok, Bialystok, Poland; Department of Regenerative Medicine, Medical University of Warsaw, Warsaw, Poland

**Keywords:** Extracellular nucleotides, Purinergic signaling, ATP, UTP, Lung cancer, Metastasis

## Abstract

**Background:**

One of the challenging problems of current radio-chemotherapy is recurrence and metastasis of cancer cells that survive initial treatment. We propose that one of the unwanted effects of radiochemotherapy is the release from damaged (“leaky”) cells of nucleotides such as ATP and UTP that exert pro-metastatic functions and can directly stimulate chemotaxis of cancer cells.

**Methods:**

To address this problem in a model of human lung cancer (LC), we employed several complementary *in vitro* and *in vivo* approaches to demonstrate the role of extracellular nucleotides (EXNs) in LC cell line metastasis and tumor progression. We measured concentrations of EXNs in several organs before and after radiochemotherapy. The purinergic receptor agonists and antagonists, inhibiting all or selected subtypes of receptors, were employed in *in vitro* and *in vivo* pro-metastatic assays.

**Results:**

We found that EXNs accumulate in several organs in response to radiochemotherapy, and RT-PCR analysis revealed that most of the P1 and P2 receptor subtypes are expressed in human LC cells. EXNs were found to induce chemotaxis and adhesion of LC cells, and an autocrine loop was identified that promotes the proliferation of LC cells. Most importantly, metastasis of these cells could be inhibited in immunodeficient mice in the presence of specific small molecule inhibitors of purinergic receptors.

**Conclusions:**

Based on this result, EXNs are novel pro-metastatic factors released particularly during radiochemotherapy, and inhibition of their pro-metastatic effects via purinergic signaling could become an important part of anti-metastatic treatment.

**Electronic supplementary material:**

The online version of this article (doi:10.1186/s12943-015-0469-z) contains supplementary material, which is available to authorized users.

## Background

There are well-known side effects of chemotherapy and radiotherapy, mainly due to the toxicity-related impaired function of vital organs [1, 2]. However, in addition, these therapies induce the unwanted expression and release of several pro-metastatic factors that create a pro-metastatic microenvironment [1, 3, 4]. Surprisingly, this issue has not been fully investigated thus far.

We propose the novel concept that toxic damage in various organs leads to an upregulation of the expression and activity of several factors in “bystander” tissues, including extracellular nucleotides (EXNs), which provide chemotactic signals to cancer cells that survived the initial treatment. We propose that this mechanism plays an important role in the metastasis of cancer cells and indicates the need to develop efficient anti-metastatic drugs that work in combination with, or follow, standard therapies in order to prevent the possibility of therapy-induced spread of tumor cells [1, 3–5].

Nucleotides leaking through the membranes of damaged cells or released through specific pathways include purine nucleotides and nucleosides (e.g., ATP, ADP, AMP, adenosine) as well as pyrimidine nucleotides (e.g., UTP, UDP), which signal through purinergic receptors expressed in almost every tissue [6]. There are both nucleoside- and nucleotide-activated receptors, which belong to different receptor families and are distinguishable by their pharmacological properties. While P1 receptors, which are divided into A_1_, A_2A_, A_2B_, and A_3_ subtypes, respond to adenosine and its analogues, P2 receptors are activated by ATP and/or other nucleotides. P2 receptors are further subdivided into ionotropic (P2X) and metabotropic (P2Y) receptors, based on structural characteristics [7, 8]. Ionotropic P2X receptors are assembled in a trimeric form as homo- or heteromers consisting of the subunits designated P2X1–7. P2X receptor channels are activated by ATP, which opens the channel to allow the influx of Ca^2+^, Na^+^, and K^+^. The mammalian metabotropic P2 receptor family contains eight different subtypes, denoted P2Y1, 2, 4, 6, 11, 12, 13, and 14 [7, 8].

Nucleotides may also be released from cells in response to certain stimulatory agents and affect the cell in an autocrine/paracrine manner. For example, the migration of leucocytes in response to analpylatoxin C5a is potentiated by the release of ATP at the leading edge of migrating cells [9]. The availability and lifetime of released ATP in a controlled manner for autocrine or paracrine stimulation of purinergic receptors is controlled by a highly efficient enzymatic cascade, including processing that degrades nucleotides (e.g. ATP, ADP, and AMP), finally yielding nucleosides (e.g. adenosine) and thereby regulating activity levels of the various P2 and P1 receptors [10]. It has been reported that, while the interstitial ATP in normal tissues attains a concentration of up to 1000 nM, the intratumoral ATP concentration can be as much as 10^3^–10^4^ fold higher [11].

Taking into consideration the possibility that EXNs affect the behavior of LC cells, we became interested in their role in progression of this tumor. We observed that EXNs accumulate in several murine organs in response to radiochemotherapy and that most of the functional P2X, P2Y, and P1 receptor subtypes are expressed in human LC cells. EXNs were found to modulate the pro-metastatic behavior of LC cells, and their metastasis could be inhibited in immunodeficient mice in the presence of specific small molecule inhibitors of purinergic receptors. Based on these findings, it is clear that EXNs are novel pro-metastatic factors and that inhibition of their pro-metastatic effects via purinergic signaling could become an important part of anti-metastatic treatment.

## Methods

### Cell lines

We used several human lung cancer cell lines (obtained from the American Type Culture Collection, Manassas, VA), including both non-small cell lung cancer (NSCLC; A549, HTB177, HTB183, and CRL5803) and small cell lung cancer (SCLC; CRL2062 and CRL5853) cell lines. NSCLC cells were cultured in Roswell Park Memorial Institute (RPMI) medium 1640, containing 10 % fetal bovine serum (FBS), 100 U/ml penicillin, and 10 μg/ml streptomycin. CRL2062 cells were maintained in Waymouth’s MB 752/1 medium containing 10 % FBS, 100 U/ml penicillin, and 10 μg/ml streptomycin. CRL5853 cells were cultured in DMEM:F12 medium supplemented with 5 % FBS, 0.005 mg/ml insulin, 0.005 mg/ml transferrin, 30 nM sodium selenite (ITS, Lonza, Allendale, NJ), 10 nM hydrocortisone (Sigma-Aldrich, St. Louis, MO, USA), 10 nM beta-estradiol (Sigma-Aldrich), 4 mM L-glutamine, 100 U/ml penicillin, and 10 μg/ml streptomycin. All cells were cultured in a humidified atmosphere of 5 % CO_2_ at 37 °C, and the media were changed every 48 h.

### Preparation of conditioned media

Pathogen-free C57BL6 mice were purchased from the National Cancer Institute (Frederick, MD, USA), allowed to adapt for at least 2 weeks, and used for experiments at age 7–8 weeks. Animal studies were approved by the Animal Care and Use Committee of the University of Louisville (Louisville, KY, USA). Mice were irradiated with 250, 500, 1000, or 1500 cGy. Twenty-four hours later, bone marrow and plasma were isolated. Conditioned medium (CM) was obtained by 1-h incubation of BM in RPMI at 37 °C. After centrifuging, the supernatant was used for further experiments. In studies with the chemotherapeutic agent vincristine, mice were injected intraperitoneally with 0.9 % NaCl with (0.5 mg/kg or 2 mg/kg) or without vincristine. Twenty-four hours later, organs were isolated, and CM was prepared as described above.

### Chemotaxis assay

Chemotaxis assays were performed in a modified Boyden’s chamber with 8-μm polycarbonate membrane inserts (Costar Transwell; Corning Costar, Lowell, MA, USA) as described previously [3, 4]. In brief, cells detached with 0.25 % trypsin were made quiescent by incubation for 1–3 h in appropriate medium (RPMI, DMEM-F12, or Waymouth’s MB 752/1), supplemented with 0.5 % (NSCLC) or 0.2 % (SCLC) bovine serum albumin (BSA). The cells were then seeded into the upper chamber of an insert (pretreated with 1 % gelatin) at a density of 3.5 × 10^4^ (NSCLC) or 10 × 10^4^ (SCLC) in 110 μl. The lower chamber was filled with pre-warmed medium containing test reagents. All nucleotides (adenosine triphosphate, ATP; adenosine diphosphate, ADP; adenosine monophosphate, AMP; adenosine; uridine triphosphate, UTP; guanosine triphosphate, GTP; thymidine triphosphate, TTP; cytidine triphosphate, CTP) were obtained from Sigma-Aldrich. Medium supplemented with BSA was used as a negative control. In some experiments, cells were pretreated with the P2 receptors inhibitor *iso*-PPADS (Tocris, Minneapolis, MN), the A_1_ receptor agonist PSB63 (Tocris), the A_2A_ receptor antagonist ANR94 (Tocris), the A_2B_ receptor antagonist PSB603 (Tocris), the A_3_ receptor antagonist MRS3777 (Tocris) or the stimulator ivermectin (Sigma-Aldrich) for 15 min at 37 °C. Inhibitors were also added to the lower chambers and were present throughout the experiment. In experiment with apyrase, apyrase (Sigma) was added to lower chamber together with HGF. After 24 h, the inserts were removed from the Transwell supports. The cells that had not migrated were scraped off with cotton wool from the upper membrane, and the cells that had transmigrated to the lower side of the membrane were fixed and stained with HEMA 3 (manufacturer’s protocol, Fisher Scientific, Pittsburgh, PA) and counted on the lower side of the membrane using an inverted microscope.

### Adhesion assay to fibronectin

Cells were made quiescent for 3 h with appropriate medium containing BSA and incubated with nucleotides for 10 min. Subsequently, cell suspensions (5 × 10^3^/100 μL) were added directly to 96-well plates coated with fibronectin and incubated for 5 min at 37 °C. The wells were previously coated with fibronectin (10 μg/ml) overnight at 4 °C and blocked with 0.5 % BSA for 1 h before the experiment. Following incubation, the plates were vigorously washed three times to remove non-adherent cells, and the number of adherent cells was counted using an inverted microscope.

### Real-time quantitative reverse-transcription PCR

Total RNA was isolated from LC cells with the RNeasy kit (Qiagen, Valencia, CA). Human lung RNA was obtained from Ambion (Austin, TX). The RNA was reverse transcribed with MultiScribe reverse transcriptase, oligo(dT), and random-hexamer primer mix (Life Techonologies, Foster City, CA). Quantitative assessment of mRNA levels was done by real-time reverse transcription PCR (qRT-PCR) on an ABI 7500 Fast instrument with Power SYBR Green PCR Master Mix reagent. Real-time conditions were as follows: 95 °C (15 s), 40 cycles at 95 °C (15 s), and 60 °C (1 min). According to melting point analysis, only one PCR product was amplified under these conditions. The relative quantity of a target, normalized to the endogenous β2-microglobulin gene as control and relative to a calibrator (normal lung tissue), is expressed as 2^−ΔΔCt^ (fold difference), where Ct is the threshold cycle, ΔCt = (Ct of target genes) − (Ct of the endogenous control gene, β2-microglobulin), and ΔΔCt = (ΔCt of lung cancer cell line sample cDNA for target gene) − (ΔCt of normal lung tissue cDNA for the target gene). All primers that were used for qRT-PCR are listed in Additional file 1: Table S1.

### Flow cytometry

For measuring A_2B_ expression cells were detached using non-enzymatic reagent (CellStripper, Corning), followed by 2 h incubation in appropriate medium with 0.5 % BSA. Next cells were washed with PBS, fixed by 15 min incubation at 4 °C in BD Cytofix/Cytoperm solution (BD Biosciences, Franklin Lakes, NJ, USA), washed again and incubated for 30 min in 0.5 % BSA in PBS. Cells were stained with primary rabbit polyclonal anti-A2B antibody (1:25, Bioss Inc, Woburn, MA, USA) for 1 h at 37 °C. Than cells were washed and incubated with secondary antibody Alexa Fluor 488 goat anti-rabbit (1:100, Life Technologies). Cells were then analyzed using the LSR cell cytometer (BD Biosciences). For all other receptors cells were detached and mechanically dissociated to a single cell suspension using TrypLE™ Express (Life Technologies) for 1 min at room temperature, passed through a 40 μm cell strainer and then washed twice with phosphate-buffered saline (PBS). Cells were subsequently fixed with 4 % (PBS) for 30 min at RT, washed and incubated for 30 min in a blocking solution containing 0.05 % Triton X-100, 0,05 % Tween-20 and 5 % FBS in PBS. Staining with primary antibodies was performed after 2 h incubation with primary antibodies: rabbit polyclonal anti-P2X4 (1:200, Santa Cruz Biotech, Dallas, TX, USA), rabbit polyclonal anti-P2X7 (1:200, Aviva Systems Biology, Corp., San Diego, CA, USA), goat polyclonal anti-P2Y1 (1:200, Santa Cruz Biotech), rabbit polyclonal anti-P2Y12 (1:500, Alomone, Jerusalem, Israel). Cells then were washed and incubated for 40 min with Alexa Fluor 488 donkey anti-rabbit (1:1,000, Life Technologies) or Alexa Fluor 488 donkey anti-goat (1:1,000, Life Technologies) secondary antibodies. Cells were analyzed with the AttuneVR cytometer (Life Technologies). The analysis of the data was performed using the FlowJo 7.2.5 or 7.6.3 software (FLOWJO, Ashland, OR, USA). Unstained cells and cells incubated with isotype control were used as controls.

Mean relative of fluorescence intensity analysis is presented as a value of mean of fluorescence intensity for stained cells divided by mean of fluorescence intensity obtained for control cells.

### Cell proliferation

Cells were seeded in culture flasks at an initial density of 1.25 × 10^4^ cells/cm^2^ (NSCLC) or 6 × 10^4^ cells/cm^2^ (SCLC). After 24 h, the medium was changed to new medium supplemented with 0.5 % BSA, and the cells were cultured in the presence or absence of nucleotides. Full medium (with FBS) was treated as a positive control. The cell number was calculated at 24, 48, and 72 h after the change of medium. At the indicated time points, cells were harvested from the culture plates by trypsinization and counted using Trypan Blue and a Neubauer chamber.

### Phosphorylation of intracellular pathway proteins

The HTB177 and CRL5803 cell lines were kept overnight or 6 h, respectively, in medium containing 0.5 % BSA to render the cells quiescent. The cells were then stimulated with nucleotides at 37 °C for 5 min, then lysed for 20 min on ice in RIPA lysis buffer containing protease and phosphatase inhibitors (Santa Cruz Biotech, Santa Cruz, CA). The extracted proteins were separated on a 12 % SDS-PAGE gel and transferred to a PVDF membrane. Phosphorylation of the serine/threonine kinase AKT (phospho-AKT473) and p44/42 mitogen-activated kinase (phospho-p44/42 MAPK) was detected by rabbit and mouse antibodies (Cell Signaling, Danvers, MA, USA), respectively, with HRP-conjugated goat anti-rabbit and anti-mouse secondary antibodies (Santa Cruz Biotech). Equal loading in the lanes was evaluated by stripping the blots and reprobing with anti-p42/44 MAPK monoclonal antibody (clone no. 9102, Cell Signaling) and anti-AKT polyclonal antibody (Cell Signaling). The membranes were developed with enhanced chemiluminescence (ECL) reagent (Amersham Life Sciences, Arlington Heights, IL), dried, and subsequently exposed to film (Hyperfilm, Amersham Life Sciences).

### Calcium measurements by microfluorimetry

Cell suspensions (5 × 10^4^/100 μL) were seeded onto a black 96-well, clear-bottom plate in appropriate medium and cultured until reaching 80–90 % confluence. The intracellular calcium concentration transient was measured using the FlexStation Calcium 4 Assay Kit (Molecular Devices Corp.), as reported elsewhere [12]. To explore the mechanism of agonist efficacy, the indirect fluorescence were determined using a FlexStation III plate reader (Molecular Devices Corp., Sunny Valley, CA). Briefly, the cells were incubated for 1 h at 37 °C with the calcium indicator solution containing 2.5 mM probenecid in a 200-μL final volume per well. The fluophore was excited at 485 nm, and the emitted fluorescence was detected at 525 nm. Changes in free intracellular calcium concentration ([Ca^2+^]_*i*_) were determined by subtracting the minimum fluorescence intensity from the maximum fluorescence intensity (Fmax–Fmin), normalized by the baseline resting state.

### Quantitation of ATP, UTP and adenosine

The ATP levels secreted by cultured LC cells were measured using the Adenosine 5′-triphosphate Bioluminescent Somatic Cell assay kit (Sigma-Aldrich), according to the manufacturer’s instructions. Briefly, cells were seeded into black 96-well microplates with transparent bottoms, and the light emitted by luciferase activity was detected using a FlexStation III plate reader (Molecular Devices Corp., Sunny Valley, CA), with the light intensity proportional to ATP concentration. The ATP level in bone marrow (BM), conditioned medium from BM, and plasma were measured using the ATP Colorimetric/Fluorometric Assay Kit and Deproteinizing Sample Preparation Kit (BioVision, Milpitas, CA, USA), according to the manufacturer protocol. Fluorescence analysis was performed with Ex/Em set at 535/585 nm. Adenosine level was measured using Adenosine Fluorymetic Assay Kit (BioVision) and UTP level was measured using UTP ELISA Kit (MyBio Source, San Diego, CA, USA) according to the manufacturers’ protocols.

Bone marrow cell lysates and conditioned media were obtained by flushing the bone marrow tibia and femur cavities and resuspending cells in 3 ml of RPMI medium. Cells suspensions were than centrifuged (680 × g, 10 min, 4 °C) and supernatants were collected and employed in experiments as conditioned media. Bone marrow cells were than washed with PBS, counted using Tuerk solution and cells pellets were fast frozen and stored in −80 °C. To obtained cell lysates frozen pellets were resuspended in 200 μl of PBS cells and subjected to ultrasonication 10 times (1 s) followed by centrifugation at 20,000 × g for 10 min, 4 °C to remove cell debris.

### Transplant of LC cells into immunodeficient mice

To study the effects of the pharmacological inhibition of P2X or A2b signaling on the metastasis of lung cancer *in vivo*, HTB177 cells were pretreated with *iso*-PPADS (100 μM), PSB603 (1 μM), or vehicle alone for 1 h. The cells were then washed and injected intravenously (2.5 × 10^6^ per mouse) into severe combined immunodeficient (SCID)-Beige inbred mice (five mice per group) that were either untreated (control) or previously irradiated with 1000 cGy for 24 h. Marrows, livers, and lungs were removed 48 h after injection of these cells, and the presence of LC cells (i.e., murine–human chimerism) was evaluated as the difference in the level of human α-satellite DNA expression. DNA was amplified in the extracts isolated from BM-, liver-, and lung-derived cells using real-time PCR. Briefly, DNA was isolated using the QIAamp DNA kit (Qiagen). Detection of human satellite and murine β-actin DNA levels was conducted using real-time PCR and an ABI Prism 7500 Sequence Detection System. A 25-μl reaction mixture containing 12.5 μl SYBR Green PCR Master Mix, 300 ng DNA template, 5′-ACC ACT CTG TGT CCT TCG TTC G-3′ forward and 5′-ACT GCG CTC TCA AAA GGA GTG T-3′ reverse primers for α-satellite DNA, and 5′-TTC AAT TCC AAC ACT GTC CTG TCT -3′ forward and 5′- CTG TGG AGT GAC TAA ATG GAA ACC -3′ reverse primers for β-actin DNA was used. The Ct value was determined as before. The number of human cells present in the murine organs (the degree of chimerism) was calculated from the standard curve obtained by mixing different numbers of human cells with a constant number of murine cells.

### Statistical Analysis

Statistical analysis of the data was done using T–test (for data with normal distribution) or Whitney–Mann test (data without normal distribution) with *p* < 0.05 considered significant or one way ANOVA with Bonferroni post hoc p ≤ 0.05 (calcium measurements and analysis of ATP in culture medium).

## Results

### The ATP level increases in response to irradiation and chemotherapy, and EXNs induce migration and adhesion of lung cancer cell lines

In our previous work we proposed that a side effect of radiochemotherapy is the induction of a pro-metastatic environment due to upregulation of several chemokines and growth factors as well as bioactive lipids [3, 4, 13]. Here we tested the hypothesis that damage caused by radiochemotherapy treatment may also release pro-metastatic EXNs from damaged “leaky” bystander cells [1]. To test this possibility, we analyzed the level of ATP in supernatants flushed from bone marrow (BM) cavities as well as in media conditioned by cultured BM cells isolated from untreated, irradiated, or vincristine-treated animals.

**Fig. 1:**
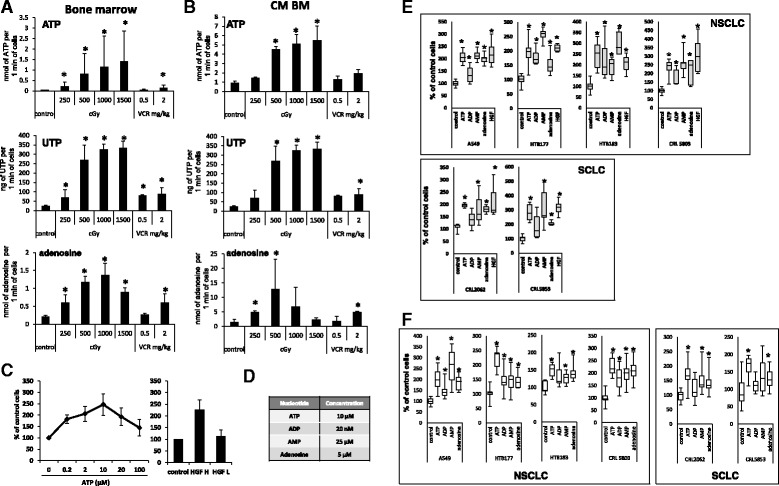
The level of EXNs increases after irradiation or chemotherapy, and EXNs induce migration and adhesion of lung cancer cell lines. Panels **a** and **b**. The level of ATP, UTP and adenosine in BM lysate (Panel **a**) and CM from BM cells (Panel **b**) prepared from animals 24 h after irradiation (0–1500 cGy) or vincristine administration (0.5–2 mg/kg). Measurements were performed for samples from three (ATP) or two (UTP and adenosine) independent isolations. Each analysis was performed in duplicates. Panel **c**. Dose-dependent chemotactic response of the HTB177 cell line to ATP (left) and hepatocyte growth factor (HGF) at physiological (L; 0.3 ng/ml) or supraphysiological (H; 10 ng/ml) concentrations (right). Panel **d**. Optimal doses of the tested nucleotides based on chemotactic dose-dependent responses of lung cancer cell lines tested with ATP, ADP, AMP, and adenosine. Panel **e**. Chemotaxis of NSCLC and SCLC cells in response to ATP, ADP, AMP, and adenosine and a supraphysiological dose of HGF (10 ng/ml). Experiment was performed at least three times. Panel **f**. Adhesion of NSCLC and SCLC cells to fibronectin after stimulation with ATP, ADP, AMP, and adenosine. Experiment was performed three times. *p < 0.05

As shown in Fig. 1a and b, we observed an increase in ATP, UTP and adenosine level in BM after irradiation or vincristine administration. Moreover, this increase in level of ATP, UTP and adenosine correlates with lower level of cells after irradiation or vincristine administration (Additional file 2: Figure S1A) suggesting that analyzed nucleotides and nucleoside are released from dying cells. Interestingly we did not observe significant changes in the level of ATP, UTP and adenosine in murine plasma (Additional file 2: Figure S1b).

Next, we tested the responsiveness of human LC cells to a chemotactic gradient of EXNs. Figure 1c shows an example of the migration of HTB177 cells in response to increasing levels of ATP (left panel) compared with high and low (physiological) concentrations of a known chemoattractant of LC cells, hepatocyte growth factor (HGF, right panel). Based on similar experiments, we established the optimal dose of EXNs, which was subsequently employed in our studies (Fig. 1d) unless otherwise indicated.

Figure 1e shows that all NSCLC cell lines responded by chemotaxis to ATP, ADP, AMP, and adenosine in a manner similar to a high dose of HGF (upper panel). We observed a similar responsiveness of SCLC cell lines to stimulation with nucleotides (lower panel). The chemotactic response of LC cells to nucleotides correlated with increased adhesion of these cells to fibronectin after stimulation with EXNs (Fig. 1f). To our surprise lung cancer cell lines turned out to be highly sensitive to ADP exposure and responded by enhanced migration and adhesion to relatively low concentration of this nucelotide (20 nM) (Fig. 1e and f). Of note, in parallel experiments we also observed that several other nucleotides, such as TTP, UTP, CTP, and GTP, chemoattract and stimulate adhesion of human LC cells (Additional file 3: Figure S2a, b).

### Lung cancer cell lines express functional P1 and P2 receptors

**Fig. 2:**
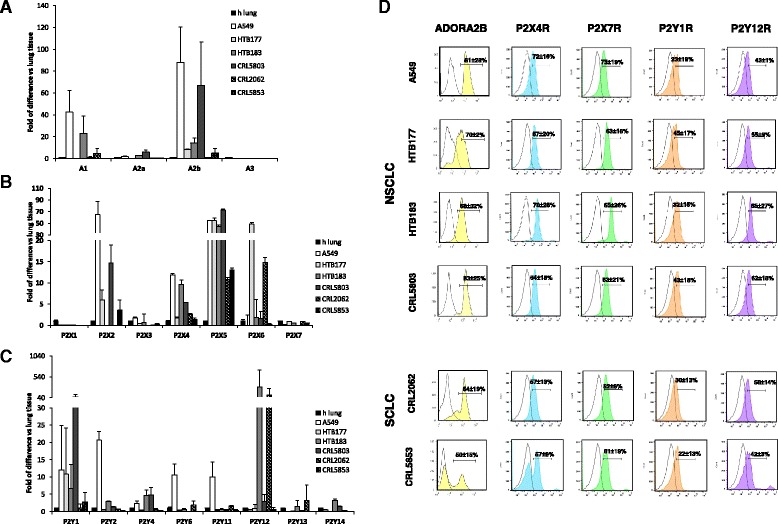
Lung cancer cell lines express functional P1 and P2 receptors. Panels **a–c**. qRT-PCR analysis of P1 (Panel **a**), P2X (Panel **b**), and P2Y (Panel **c**) receptors in human lung cancer cell lines. Normal lung tissue RNA (h lung) was used for comparison. Analysis has been performed twice in duplicates. Panel **d**. Flow Cytometry analysis of A_2B_, P2X4, P2X7, P2Y1, P2Y12 receptors

Since EXNs signal through cell membrane receptors, we employed real time-PCR to assess the expression of P1 and P2 receptors in the NSCLC and SCLC cell lines employed in our studies. We observed that, out of all the P1 receptors, the NSCLC cell lines express the adenosine A_2B_ receptor at a high level, although it is not expressed by the SCLC cell lines (Fig. 2a). Most of the LC cell lines evaluated in our study also expressed the P2X (Fig. 2b) and P2Y receptors (Fig. 2c). In particular, the P2X4 and P2X5 receptors were expressed by all cell lines employed in our studies. To confirm presence of receptors on cell surface, we performed flow cytometry analysis of A_2B_, P2X4, P2X7, P2Y1 and P2Y12 receptors and we were able to detect expression of analyzed receptors in all tested cell lines (Fig. 2d). We performed also mean relative of fluorescence intensity (MRFI) analysis (Additional file 4: Figure S3) of receptor expression. Level of receptor expression by FACS corresponded in majority of cases with our qRT-PCR data (Fig. 2a-c). However, as it is known expression of given protein at mRNA level does not always correlate with translated protein. Therefore, some of differences in expression level an RNA and protein level observed could be explained for example by different turnover and half-life of the mRNA and corresponding protein in different cell lines.

**Fig. 3:**
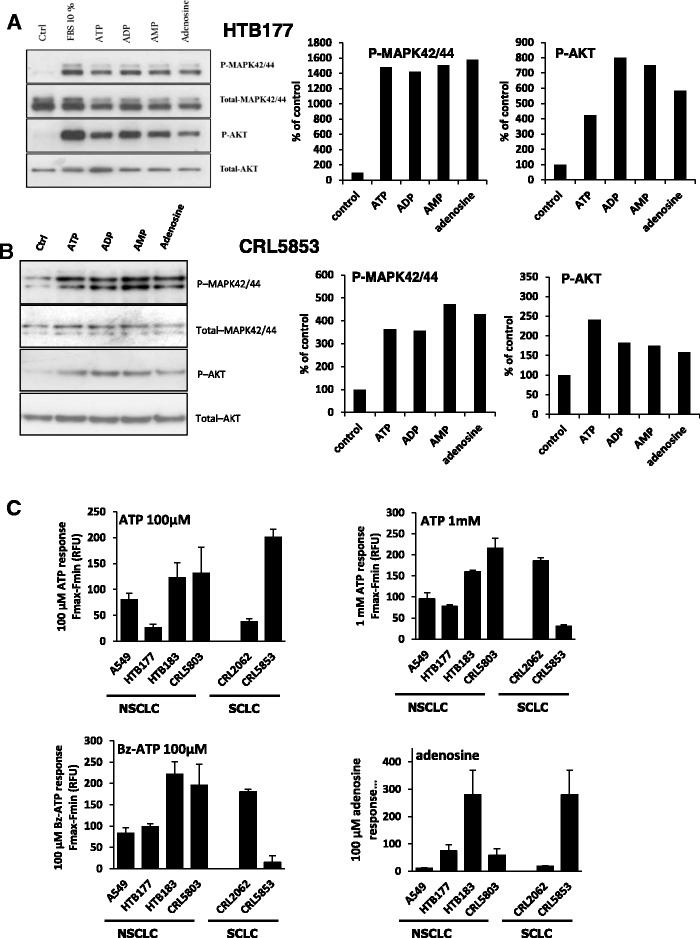
P1 and P2 receptors are functional in lung cancer cell lines. Panels **a**-**b**. Phosphorylation of p42/44 MAPK and AKT in the human NSCLC cell line HTB177 (Panel **a**) and the SCLC cell line CRL5853 (Panel **b**) after being stimulated for 5 min with the indicated nucleotides. The experiment was repeated twice, with similar results, and representative western blots are shown. Right panels show quantitative analysis of MAPKp42/44 and AKT phosphorylation. Panel **c**. Intracellular calcium measurments in lung cancer cell lines in response to 100 μM ATP (left top) or 1 mM ATP (right top), 100 μM Bz-ATP (left bottom), or 100 μM adenosine (right bottom). Analysis was performed at least 3 times in triplicates. Values are mean ± SEM

To better address the functionality of purinergic receptors, we examined whether purinergic nucleotides and nucleosides induce signaling pathways involved in cell migration and adhesion [14, 15]. As shown in Fig. 3 panels a and b, ATP, ADP, AMP, and adenosine induced MAPK p42/44 and AKT phosphorylation in the NSCLC cell line HTB177 and the SCLC cell line CRL5853. The left side of these figures shows a western blot, and the right side shows the corresponding densitometry measurements. Of note, we also observed that other nucleotides, such as UTP, CTP, GTP, and TTP, induced phosphorylation of MAPKp42/44 and AKT in HTB177 and CRL5853 LC cell lines (Additional file 5: Figure S4).

Next, since P2X and P2Y receptor stimulation may result in [Ca^2+^]_*i*_ increase, we measured whether LC cells show calcium concentration transients in response to P2 receptor agonist ATP and Bz-ATP, which is a P2X7 receptor agonist that is 5–30 times more potent than ATP and can also stimulate all P2X receptors. We found that all cell lines tested responded by calcium signaling upon stimulation by ATP (Fig. 3c upper panel) as well as by Bz-ATP (Fig. 3c lower left panel), and their responsiveness varied with the cell line tested. Interestingly, while expression of the P2X7 receptor was low in LC cell lines (Fig. 2b), Bz-ATP turned out to be a potent stimulator of calcium signaling, probably due to stimulation of all P2X receptors. Of note, UTP, a P2Y2 and P2Y4 receptor agonist, also stimulated intracellular calcium mobilization (Additional file 3: Figure S2c). As shown in the lower right panel of Fig. 3c, adenosine also induced intracellular calcium fluxes in human LC cell lines. All these data confirm that human lung cancer cells express functional purinergic receptors.

### Small molecule inhibitors of purinergic receptors modulate the chemotactic responsiveness of LC cells in a receptor-dependent manner

**Fig. 4:**
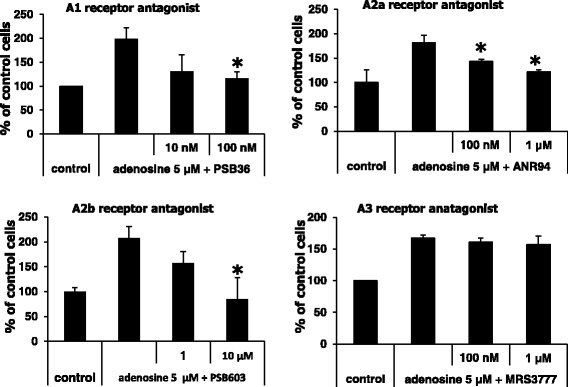
P1 receptors regulate the migratory properties of lung cancer cells. The effect of adenosine receptor inhibitors on the migration of A549 cells. Migration of cells acros Transwell membrane in response to adenosine in the presence of PSB36 (an A1 receptor antagonist), ANR 94 (an A2a receptor antagonist), PSB603 (an A2b receptor antagonist), and MRS3777 (an A3 receptor antagonist). The experiment was repeat three times with similar results. All values are mean ± SD with *p < 0.05

To test the efficacy of small molecule inhibitors of P1 receptor signaling in LC cells, we tested the effect of different P1 receptor inhibitors using the A549 cell line, which expresses adenosine A_1_, A_2A_, and A_2B_ receptors at the highest levels of all the analyzed cell lines but not the A_3_ receptor (Fig. 2a) as an experimental model (Fig. 4). We found that A_1_ (PSB36), A_2A_ (ANR94), and, in particular, A_2B_ (PSB603) receptor antagonists partially inhibited migration of A549 cells in response to adenosine, which is a P1 receptor agonist. Of note, the A_2B_ receptor was found to be highly expressed by these cells. At the same time, as expected, since the A_3_ receptor is not expressed by A549 cells, we did not observe any effect on the migration of these cells across Transwell membranes in response to adenosine in the presence of the A_3_ receptor antagonist MRS3777 (Fig. 4 lower right panel). Interestingly, we also found that sensitivity of LC cells to PSB603 is correlated with the level of expression of A_2B_ receptor. Accordingly, inhibition of migration of HTB177 cells which express lower level of A_2B_ receptor than A549 was already observed in presence of 1 μM PSB603 (data not shown).

**Fig. 5:**
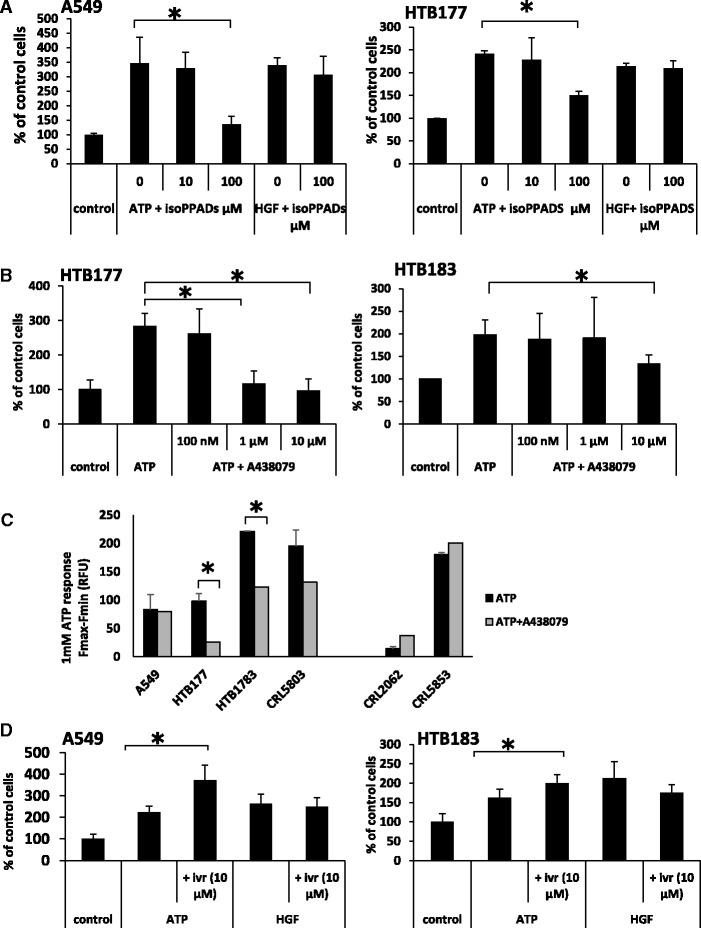
P2 receptors regulate the migratory properties of lung cancer cells. Panel **a** The effect of *iso*-PPADS on the migration of A549 (left) and HTB177 (right) cells across Transwell membranes in response to adenosine. HGF (10 ng/ml) was used as positive control. The experiment was done three times. *p < 0.05. Panel **b**. The effect of the P2X7R inhibitor A438079 on the migration of HTB177 (left) and HTB183 (right) cells acros Transwell membrane in response to ATP. The experiment was done three times. *p < 0.05. Panel **c**. The effect of the P2X7 inhibitor A438079 on intracellular calcium measurements in lung cancer cell lines in response to 100 μM ATP. Analysis was performed at least 3 times in triplicates *p < 0.05. Panel **d**. The effect of pretreatment of cells with ivermectin (10 μM) on Transwell membrane migration in response to ATP. The experiment was done three times. *p < 0.05

Based on our observation that the P2X receptors are involved in migration of breast cancer [16], we became interested in whether these receptors play a role in the migration of LC cells. At first, we used a nonspecific antagonist of all P2X receptors, *iso*-PPADS, and observed its inhibitory effect on the chemotaxis of A549 and HTB177 cells in response to the P2X agonist, ATP (Fig. 5a). At the same time, in control experiments we did not observe any reduction in the chemotactic response of LC cells to HGF, which confirmed inhibitor specificity (Fig. 5a).

Since, as reported in the literature, the P2X7 receptor stimulates cell migration [17, 18], we employed an antagonist of the P2X7R, A438079, in chemotaxis assays of LC cell lines in response to ATP. As shown in Fig. 5b, A438079 inhibited migration of HTB177 and HTB183 in a P2X7-dependent manner. This result corroborated the results of the intracellular calcium release experiments (Fig. 5c) as did the P2X7 expression level (Fig. 2b).

Next, since, as shown in Fig. 2b, P2X4 receptors is expressed at the highest level in LC cell lines compared with other P2X receptors, we tested its responsiveness to an ATP gradient in the presence of ivermectin, which increases the response of the P2X4 receptor to ATP stimulation, as previously described [19, 20]. Figure 5d shows that A549 and HTB183 cells, which show the highest expression of P2X4, respond more robustly to an ATP gradient in the presence of ivermectin. At the same time, as expected in control experiments, ivermectin did not affect the migration of LC cells in response to HGF.

### Autocrine release of nucleotides can play a role in the migration of cells in response to HGF and stimulate their proliferation

Based on findings in the literature [21–23], we tested whether EXNs affect proliferation of LC cells. However, to our surprise, none of the tested nucleotides was able to stimulate or inhibit proliferation of these cells at concentrations sufficient to stimulate their migration (data not shown). On the other hand, it has been reported that nucleotides may be released from cells in response to certain factors (e.g., the complement cascade cleavage fragment C5a) and stimulate the cells’ responsiveness to autocrine/paracrine signaling axes [9].

**Fig. 6:**
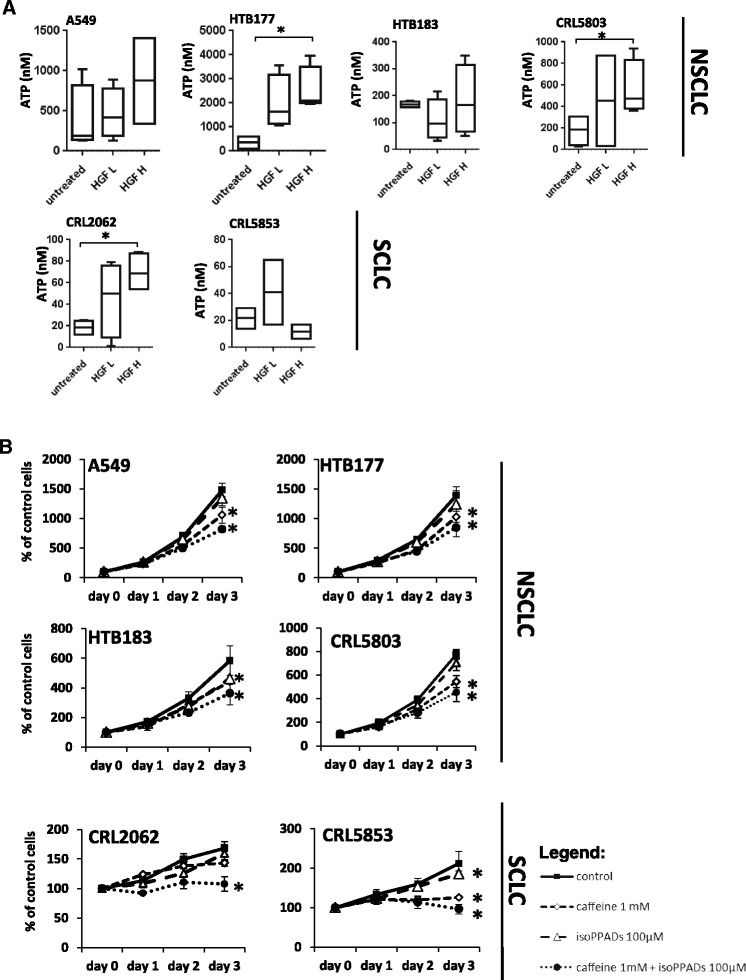
The involvement of HGF-mediated release of nucleotides from LC cells and the role of autocrine ATP loops in LC cell proliferation. Panel **a**. ATP levels in conditioned media from control untreated cells or cells treated with a low (0.3 ng/ml) or high (10 ng/ml) doses of HGF. Analysis was performed at least 3 times in triplicates *p < 0.05. Panel **b**. Proliferation of lung cancer cells in the presence of the adenosine receptor inhibitor caffeine and the P2X receptor inhibitor *iso*-PPADS. The experiment was performed three times in triplicate. *p < 0.05

As shown in Fig. 6a, we were able to detect ATP in conditioned media harvested from all tested LC cell lines. Interestingly, we also found that HGF, which is a chemoattractant for LC cells, enhanced secretion of ATP by LC cells. This observation requires further study to determine whether some of the migratory effects of peptide-based chemoattractants could be related to the costimulatory effect of autocrine-secreted nucleotides, as reported for the C5a effect on leucocyte migration [9]. To check whether increased migration in response to HGF might be related to ATP release, we performed chemotaxis assay in the presence or absence of apyrase, which hydrolyzes ATP. We found that migration of HTB177 to HGF was inhibited when apyrase was added to the lower chamber (Additional file 6: Figure S5), which confirms potential role of ATP in HGF induced migration of lung cancer cells.

Finally, taking into consideration the potential effect of autocrine EXN axes in the proliferation of LC cells, we exposed LC cells to the P2X inhibitor *iso*PPADs and to caffeine, a non-specific inhibitor of the P1 receptors, alone or in combination (Fig. 6b). We found an inhibitory effect of the inhibition of P1 and P2X signaling on the proliferation of LC cells, which was most pronounced for the SCLC cell lines.

### Pretreatment of LC cells with antagonist of A_2B_ or P2X receptor decreases adenosine- and ATP-dependent metastatic spread of lung cancer cells

**Fig. 7:**
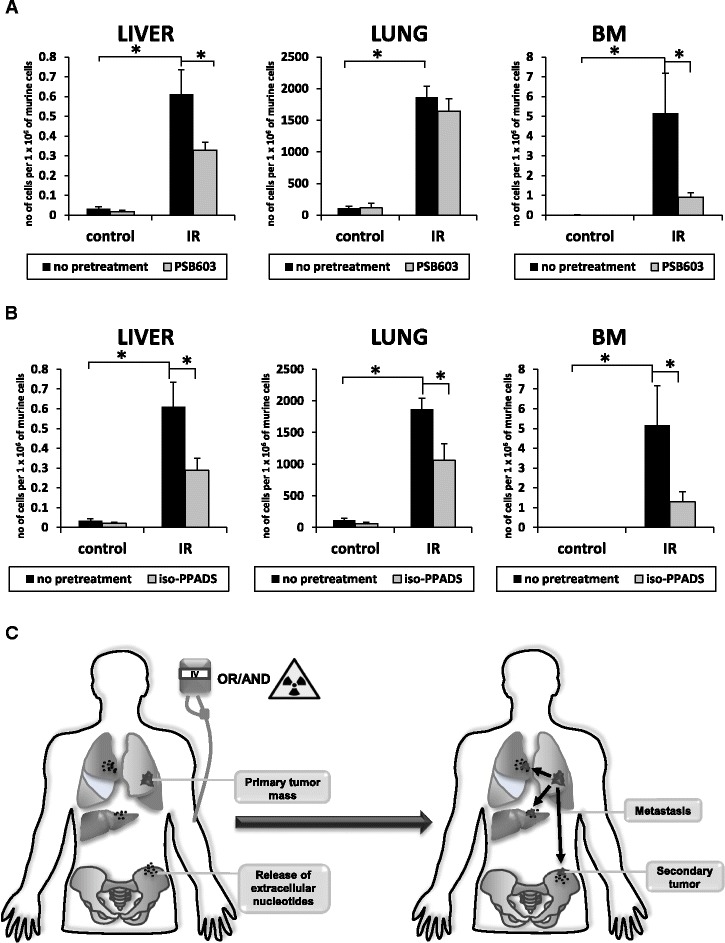
External nucleotide levels create a pro-metastatic microenvironment in irradiated organs. Panel **a**. Detection of human lung cancer cells (HTB177) in liver, lung, and BM of mice irradiated and then inoculated with LC cells. HTB177 cells before transplant were pretreated with PSB603 (1 μM) or vehicle. Five mice were employed per group, and results are shown as means ± SEM. *p < 0.05. Panel **b**. Detection of human lung cancer cells (HTB177) in liver, lung, and BM of mice irradiated and then inoculated with LC cells. HTB177 cells before transplant were pretreated with *iso-*PPADS (10 μM) or vehicle. Five mice were employed per group, and results are shown as means ± SEM. *p < 0.05. Panel **c**. Radiochemotherapy induces release of EXNs that may be reponsible for the metastatic spread of LC cells that survived the initial tretament

Finally, we tested whether inhibition of A_2B_ receptor in HTB177 cells by PSB603 affects the metastatic spread (seeding efficiency) of these cells *in vivo* to tissues damaged by irradiation. To address this issue, HTB177 cells were exposed to PSB603 for 1 h, washed, and injected into control non-irradiated and 1000-cGy-irradiated SCID/beige immunodeficient mice (Fig. 7a). We found that irradiation increases the seeding efficiency of HTB177 cells to liver, lung, and BM and that this effect was significantly decreased in the case of liver and BM after pretreatment of HTB177 cells with PSB603 (Fig. 7a). A similar experiment was performed with cells pretreated with the P2X inhibitor *iso-*PPADS. We observed significantly reduced seeding of HTB177 cells pretreated with *iso*-PPADS (Fig. 7b), which corroborates the observation that the ATP level is highly elevated in irradiated tissues (Fig. 1a,b).

## Discussion

One of the most challenging clinical problems is the tumor recurrence and metastasis of cancer cells that survive standard treatment [1, 13, 24]. To explain these phenomena, we have proposed that one of the unwanted side effects of radiochemotherapy is the induction of a pro-metastatic microenvironment in normal tissues that are damaged by the treatment, due to an increase in certain peptide- and lipid-based chemottractants [1, 3, 4, 13]. In parallel, we have hypothesized that “leaky” cells damaged by radiochemotherapy also release nucleotides that, as demonstrated in the past, are potent chemotactic factors for both normal [9, 25] and malignant cells [16, 18].

In support of a role of purinergic signaling in cancerogenesis, it is well known that malignant tumors promote strong inflammatory reactions together with necrosis, and nucleotides may leak from damaged cells into the extracellular medium or even be released by specific pathways as part of tumor cell metabolism and anti-tumor protection mechanisms [26–29]. What is also important, nucleotides may be released from the damaged cells in response to radiochemotherapy, as shown in this paper. In fact, we found an increase in ATP level in irradiated murine tissues, including BM and liver, which are known sites for cancer metastasis. It has also been reported that nucleotides may be released from cells stimulated by the fifth complement cleavage fragment, the anaphylotoxin C5a [9], and it is well known that the complement cascade is activated in cancer patients [30, 31]. In our studies, we found that HGF may also increase the secretion of ATP from LC cells, and HGF, along with C5a, is upregulated in response to radiochemotherapy [13, 32]. The question remains whether, in addition to C5a and HGF, other factors that are released in tissues in response to anti-cancer treatment such as certain chemokines [13] or bioactive lipids [3, 4] also increase the release of nucleotides from target cells, but this requires further studies.

In our work, we focused mainly on the biological effects of ATP, ADP, AMP, and adenosine, which are well-established ligands for G-protein coupled P1 and P2Y receptors and ligand-gated ion channel P2X receptors [23]. While P1 receptors are activated by adenosine and A_1_ receptor subtypes also by AMP [33], P2X receptors are activated by ATP, and P2Y receptors respond to ATP, ADP and UTP [34]. In our studies, we demonstrated that all of these nucleotides stimulate human LC cells. We also found responsiveness of LC cells to TTP, CTP, and GTP. Despite some suggestions that these nucleotides may also stimulate some P2X receptors, we cannot exclude that observed effect is due to receptor independent cell stimulation. As support of such hypothesis, similar phenomenons were already described for ATP [11] and adenosine [35]. However, we focused our current work on the most relevant EXNs and nucleoside, which are ATP, ADP, AMP, and adenosine.

We learned that human NSCLC and SCLC cells express several functional purinergic receptors. Stimulation by EXNs promoted migration and adhesion of LC cells. These functional responses of LC to nucleotides are supported by the activation of intracellular pathways, including MAPKp42/44 and AKT phosphorylation, as well as [Ca^2+^]_*i*_ transients.

Nucleotides have already been reported to stimulate proliferation of some malignant cells, including colon adenocarcinoma and melanoma cells [36, 37]. To our surprise, however, we found that, if added to LC cell cultures, nucleotides did not stimulate their growth. On the other hand, we detected ATP in conditioned media harvested from LC cells, and inhibition of purinergic signaling in these cells by *iso*-PPADS and caffeine negatively affected their proliferation. This suggests the involvement of autocrine signaling axes in the proliferation of LC cell lines. In support of a role for autocrine purinergic signaling in regulating the biology of LC cells, autocrine signaling via release of ATP and activation of the P2X7 receptor was found by another group to enhance the motility of human LC cells [17].

Nevertheless, data on the effect of nucleotides and nucleosides on the proliferation of LC cells are somewhat controversial. For example, it has been reported that treatment of A549 cells with adenosine results in their senescence, both *in vitro* and *in vivo,* through induction of cell cycle arrest and senescence in a p53/p21-dependent manner [38]. A similar effect has been observed after exposure of the PC14 lung adenocarcinoma cell line to ATP [39]. However, in another report, ATP stimulation of P2Y receptors increased the proliferation of human lung epithelial tumor cells [21]. These differences may be explained by the much higher concentrations of ATP employed in those studies compared with the concentrations that we used in our work.

It is well known that EXNs may affect different aspects of LC biology. For example, ATP was found to sensitize LC cells to cisplatin-induced apoptosis [40] and enhance the antitumor effect of etoposide in PC14 and A549 LC cells [39]. Moreover, it has been reported that extracellular ATP may be internalized by cancer cells by micropinocytosis, which induces an increase in intracellular ATP and drug resistance [11]. It was also shown that ATP- or UTP-mediated activation of P2Y2 induced cancer cell invasion through increased production of VEGF by cancer cells [41] and that adenosine receptors have been found to regulate VEGF expression under hypoxic environment in different tissues [42]. On the other hand, EXNs are potent chemoattractants for mesenchymal stromal cells and thus may attract these cells and promote stromalization of the growing tumor [43]. Similarly, EXNs may exert an effect on endothelial progenitors and thereby promote tumor vascularization [44]. Altogether, given these data, purine and pyrimidine nucleotides can be considered crucial orchestrators of both directly and indirectly regulated pro-metastatic potential of tumor cells.

What is most important in our report is that, by employing pro-metastatic assays *in vitro* and *in vivo*, we have demonstrated for the first time that purinergic signaling may be an attractive target for small molecule antagonists of purinergic receptors to inhibit the metastatic spread of LC cells. Our results employing receptor antagonists lend further support to this concept. On the other hand, it is known that degradation of EXNs in the extracellular space is regulated by enzymatic cascades, including ectonucleoside triphosphate diphosphohydrolases (E-NTPDase 1, also known as CD39; E-NTPDases 2, 3, and 8), ectonucleotide pyrophosphatases/phosphodiesterases (E-NPPs), ecto-alkaline phosphatases, and ecto-5′-nucleotidase (also known as CD73), which degrade nucleotides (e.g. ATP, ADP, and AMP), finally yielding nucleosides (e.g. adenosine) and thereby regulating activity levels of the various P2 and P1 receptors [45–47]. In addition to purinergic receptors, these enzymes are potential targets for small molecule inhibitors to control migration and metastasis of LC cells. This is of particular importance, since, as mentioned above, the concentration of EXNs in tumor tissue could be very high [11, 48]. However, the evidence supporting a functional role of ectonucleotidases in purinergic signaling varies considerably between enzyme species and thus should be taking into consideration when looking for possible anti-cancer targets [46, 47].

We are aware that the results that we generated with established human LC cell lines need to be verified with LC patient primary cells. It will be important to establish whether the pattern of purinergic receptor expression has prognostic value and whether it correlates with more malignant and metastatic phenotypes.

## Conclusion

EXNs are novel pro-metastatic factors released during radiochemotherapy (Fig. 1a and b), and inhibition of their pro-metastatic effects, which are mediated by purinergic signaling, could become a novel and important part of anti-metastatic treatment. The results reported here for LC cell lines may also be relevant for other cancers, as EXNs have been reported to stimulate several other types of cancer cells [16, 18, 49].

## Additional files

Additional file 1: Table S1. Sequences of primers used for qRT-PCR. (DOCX 15 kb) Additional file 2: Figure S1. Evaluation of the number of BM cells and the level of EXN in plasma after irradiation or chemotheraphy. Panel A Total number of cells isolated from tibia and femurs of animals 24 h after irradiation (0–1500 cGy) or vincristine administration (0.5–2 mg/kg). Combine results from three independent isolations. Panel B The level of ATP, UTP and adenosine in murine plasma isolated from animals 24 h after irradiation (0–1500 cGy) or vincristine administration (0.5–2 mg/kg). (PDF 229 kb) Additional file 3: Figure S2. Extracellular nucleotides induce migration and adhesion of lung cancer cell lines. Panel A. Chemotaxis of tested lung cancer cell lines in response to extracellular TTP, UTP, CTP, and GTP. Chemotaxis in response to a supraphysiological dose of HGF (10 ng/ml) was used as a control. Panel B. Adhesion of tested lung cancer cell lines to fibronectin after stimulation with extracellular TTP, UTP, CTP, and GTP. Panel C. UTP stimulates intracellular calcium release in human lung cancer cells lines. All data are shown as means ± SD with **p* < 0.05. (ZIP 296 kb) Additional file 4: Figure S3. Lung cancer cell lines express P1 and P2 receptors. Analysis of mean relative of fluorescence intensity of A2B, P2X4, P2X7, P2Y1, P2Y12 receptors expression obtained by Flow Cytometry. (PDF 211 kb) Additional file 5: Figure S4. Extracellular TTP, UTP, CTP, and GTP stimulate human lung cancer cells. Phosphorylation of p42/44 MAPK and AKT in the human NSCLC cell line HTB177 (Panel A) or the SCLC cell line CRL5853 (Panel B) stimulated for 5 min by the indicated nucleotides. The experiment was repeated twice, with similar results, and representative western blots are shown. (PDF 177 kb) Additional file 6: Figure S5. Autocrine release of ATP plays a role in migration of lung cancer cells to HGF. Chemotaxis results of HTB177 cell line to HGF (10 ng/ml) in the absence or presence of apyrase (50U/ml). (PDF 87 kb)
