# Supplementary material for: Extracellular nucleotides as novel, underappreciated pro-metastatic factors that stimulate purinergic signaling in human lung cancer cells
Source: Mol Cancer. 2015 Nov 24;14:201. doi: 10.1186/s12943-015-0469-z (PMC4657356; doi:10.1186/s12943-015-0469-z)
Supplement: Additional file 1: Table S1. — Sequences of primers used for qRT-PCR. (DOCX 15 kb) [file 12943_2015_469_MOESM1_ESM.docx]

Table 1. Sequences of primers used for qRT-PCR

| **Forward Primers** | **Sequence** | **Revers primers** | **Sequence** |
| --- | --- | --- | --- |
| A1_F | TGCGAGTTCGAGAAGGTCATC | A1_R | GAGCTGCTTGCGGATTAGGTA |
| A2a_F | CGAGGGCTAAGGGCATCATTG | A2a_R | CTCCTTTGGCTGACCGCAGTT |
| A2b_F | CTGTGTCCCGCTCAGGTATAA | A2b_R | ACTGTTCCACCCCAGGAATG |
| A3_F | TACATCATTCGGAACAAACTC | A3 | GTCTTGAACTCCCGTCCATAA |
| P2X1_F | CGCCTTCCTCTTCGAGTATGA | P2X1_R | AGATAACGCCCACCTTCTTATTACG |
| P2X2_F | GCCTACGGGATCCGCATT | P2X2_R | TGGTGGGAATCAGGCTGAAC |
| P2X3_F | GCTGGACCATCGGGATCA | P2X3_R | GAAAACCCACCCTACAAAGTAGGA |
| P2X4_F | CCTCTGCTTGCCCAGGTACTC | P2X4_R | CCAGGAGATACGTTGTGCTCAA |
| P2X5_F | CTGCCTGTCGCTGTTCGA | P2X5_R | GCAGGCCCACCTTCTTGTT |
| P2X6_F | AGGCCAGTGTGTGGTGTTCA | P2X6_R | TCTCCACGGGGCACCAACTC |
| P2X7_F | AGTGCGAGTCCATTGTGGAG | P2X7_R | CGCAGGTCTTGGGACTTCTT |
| P2Y1_F | CGTGCTGGTGTGGCTCATT | P2Y1_R | GGACCCCGGTACCTGAGTAGA |
| P2Y2_F | CACCCGCACCCTCTACTACT | P2Y2_R | CCTTGTAGGCCATGTTGATG |
| P2Y4_F | CCGTCCTGTGCCATGACA | P2Y4_R | GCTGAAGTGCACATAGTGGTCAA |
| P2Y6_F | GGTGCGGTCCTCAGTGAGCC | P2Y6_R | CGCCAGCACCGCCGAATACA |
| P2Y11_F | GGCTGAGGATCGGCACGGGA | P2Y11_R | ATGGGCCACAGGAAGTCCCCC |
| P2Y12_F | AGGTCCTCTTCCCACTGCTCTA | P2Y12_R | CATCGCCAGGCCATTTGT |
| P2Y13_F | GAGACACTCGGATAGTACAGCTGGTA | P2Y13_R | GCAGGATGCCGGTCAAGA |
| P2Y14_F | TCTTCATTGCAGGAATCCTACTCA | P2Y14_R | AGAGCTGGGCACGTAAAAGAAT |
| β-macro-globulin | TGACTTTGTCACAGCCCAAGATA | β-macro-globulin | AATGCGGCATCTTCAAACCT |
