# Supplementary material for: Extracellular nucleotides as novel, underappreciated pro-metastatic factors that stimulate purinergic signaling in human lung cancer cells
Source: Mol Cancer. 2015 Nov 24;14:201. doi: 10.1186/s12943-015-0469-z (PMC4657356; doi:10.1186/s12943-015-0469-z)
Supplement: Additional file 2: Figure S1. — Evaluation of the number of BM cells and the level of EXN in plasma after irradiation or chemotheraphy. Panel A Total number of cells isolated from tibia and femurs of animals 24 h after irradiation (0–1500 cGy) or vincristine administration (0.5–2 mg/kg). Combine results from three independent isolations. Panel B The level of ATP, UTP and adenosine in murine plasma isolated from animals 24 h after irradiation (0–1500 cGy) or vincristine administration (0.5–2 mg/kg). (PDF 229 kb) [file 12943_2015_469_MOESM2_ESM.pdf]

Supplementary Figure 1

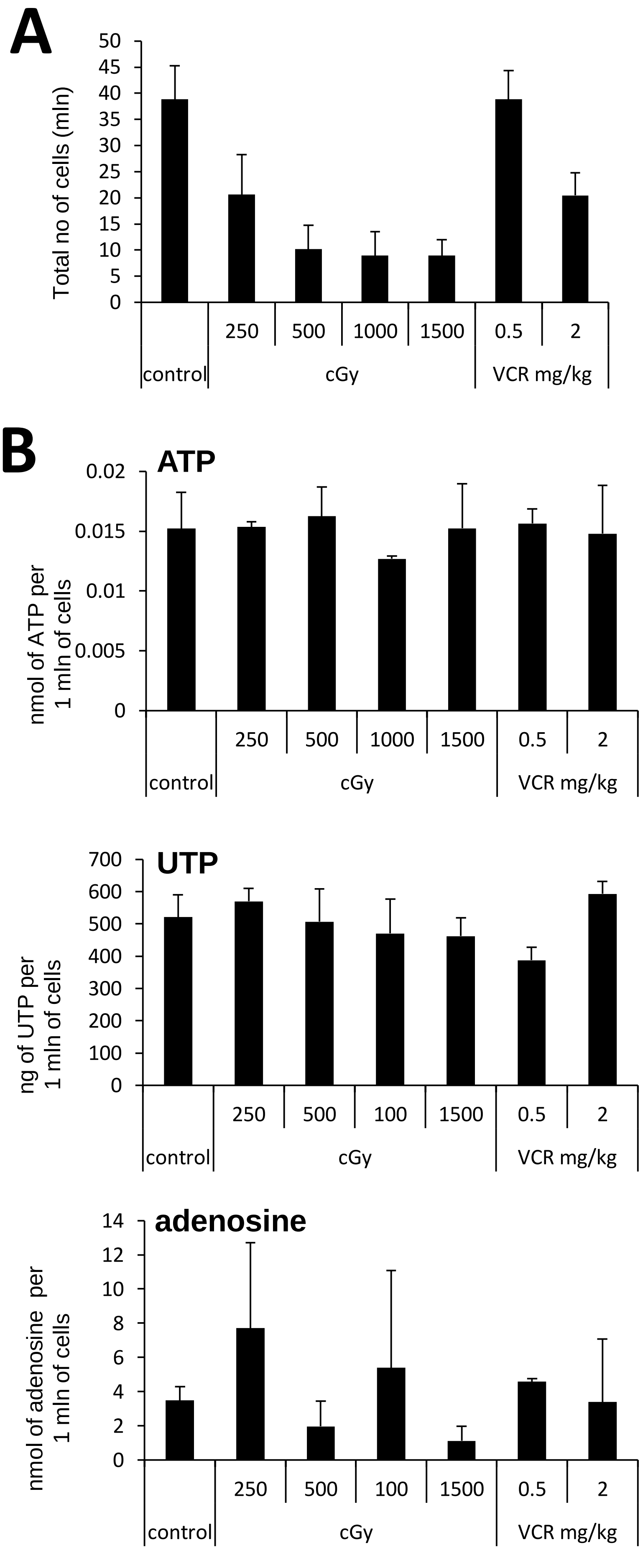

Supplementary Figure 1. **Panel A** Total number of cells isolated from tibia and femurs of animals 24 h after irradiation (0–1500 cGy) or vincristine administration (0.5–2 mg/kg). Combine results from three independent isolations.
Panel B The level of ATP, UTP and adenosine in murine plasma isolated from animals 24 h after irradiation (0–1500 cGy) or vincristine administration (0.5–2 mg/kg).
