# Supplementary material for: Extracellular nucleotides as novel, underappreciated pro-metastatic factors that stimulate purinergic signaling in human lung cancer cells
Source: Mol Cancer. 2015 Nov 24;14:201. doi: 10.1186/s12943-015-0469-z (PMC4657356; doi:10.1186/s12943-015-0469-z)
Supplement: Additional file 3: Figure S2. — Extracellular nucleotides induce migration and adhesion of lung cancer cell lines. Panel A. Chemotaxis of tested lung cancer cell lines in response to extracellular TTP, UTP, CTP, and GTP. Chemotaxis in response to a supraphysiological dose of HGF (10 ng/ml) was used as a control. Panel B. Adhesion of tested lung cancer cell lines to fibronectin after stimulation with extracellular TTP, UTP, CTP, and GTP. Panel C. UTP stimulates intracellular calcium release in human lung cancer cells lines. All data are shown as means ± SD with *p < 0.05. (ZIP 296 kb) [file 12943_2015_469_MOESM3_ESM.zip › Supplementary Figure 2C.pdf]

Supplementary Figure 2

C

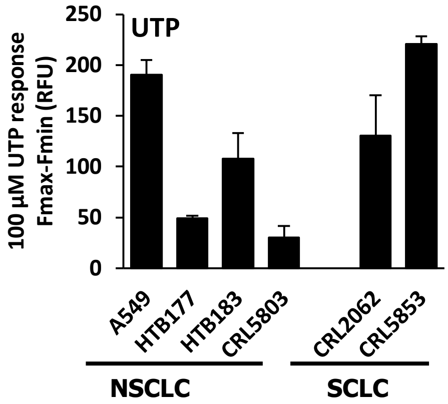

Supplementary Figure 2. Extracellular nucleotides induce migration and adhesion of lung cancer cell lines. **Panel C.** UTP stimulates intracellular calcium release in human lung cancer cells lines. All data are shown as means ± SD with *p<0.05.
