# Supplementary material for: Extracellular nucleotides as novel, underappreciated pro-metastatic factors that stimulate purinergic signaling in human lung cancer cells
Source: Mol Cancer. 2015 Nov 24;14:201. doi: 10.1186/s12943-015-0469-z (PMC4657356; doi:10.1186/s12943-015-0469-z)
Supplement: Additional file 4: Figure S3. — Lung cancer cell lines express P1 and P2 receptors. Analysis of mean relative of fluorescence intensity of A2B, P2X4, P2X7, P2Y1, P2Y12 receptors expression obtained by Flow Cytometry. (PDF 211 kb) [file 12943_2015_469_MOESM4_ESM.pdf]

Supplementary Figure 3

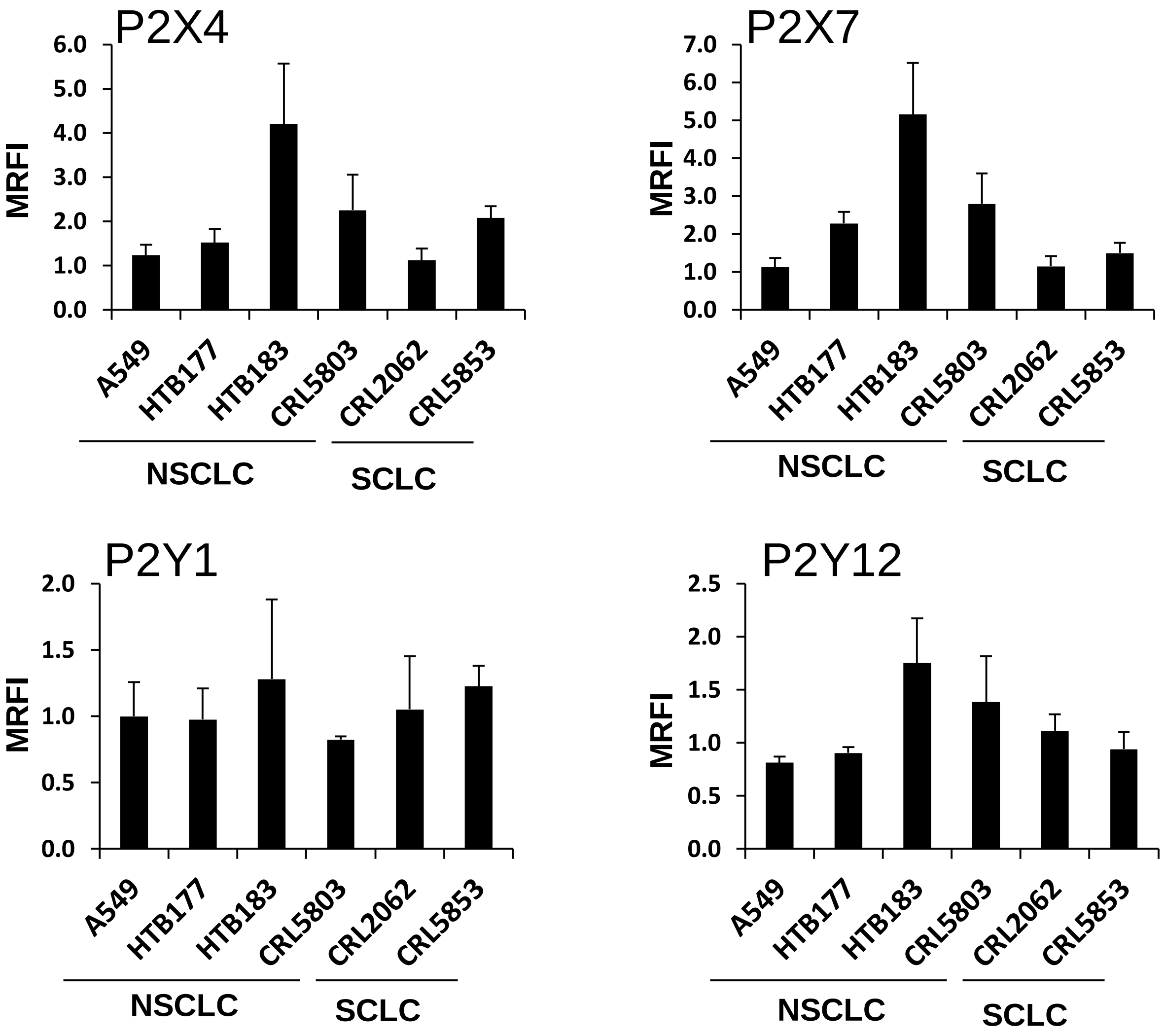

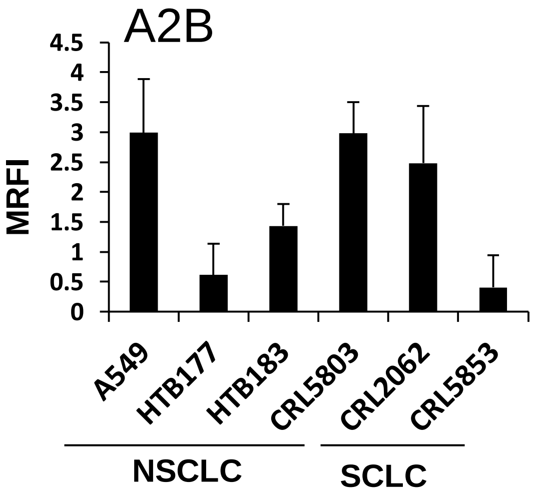

Supplementary Figure 3. . **Lung cancer cell lines express P1 and P2 receptors**. Analysis of mean relative of fluorescence intensity of A_{2B}, P2X4, P2X7, P2Y1, P2Y12 receptors expression obtained by Flow Cytometry.
