# Supplementary material for: Extracellular nucleotides as novel, underappreciated pro-metastatic factors that stimulate purinergic signaling in human lung cancer cells
Source: Mol Cancer. 2015 Nov 24;14:201. doi: 10.1186/s12943-015-0469-z (PMC4657356; doi:10.1186/s12943-015-0469-z)
Supplement: Additional file 5: Figure S4. — Extracellular TTP, UTP, CTP, and GTP stimulate human lung cancer cells. Phosphorylation of p42/44 MAPK and AKT in the human NSCLC cell line HTB177 (Panel A) or the SCLC cell line CRL5853 (Panel B) stimulated for 5 min by the indicated nucleotides. The experiment was repeated twice, with similar results, and representative western blots are shown. (PDF 177 kb) [file 12943_2015_469_MOESM5_ESM.pdf]

Supplementary Figure 4

A

HTB177

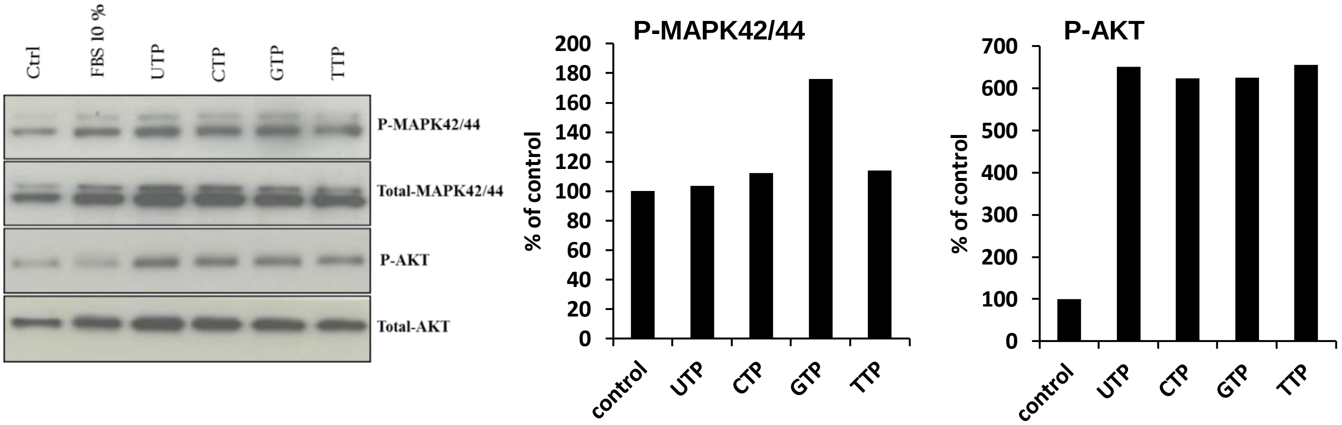

B

CRL5853

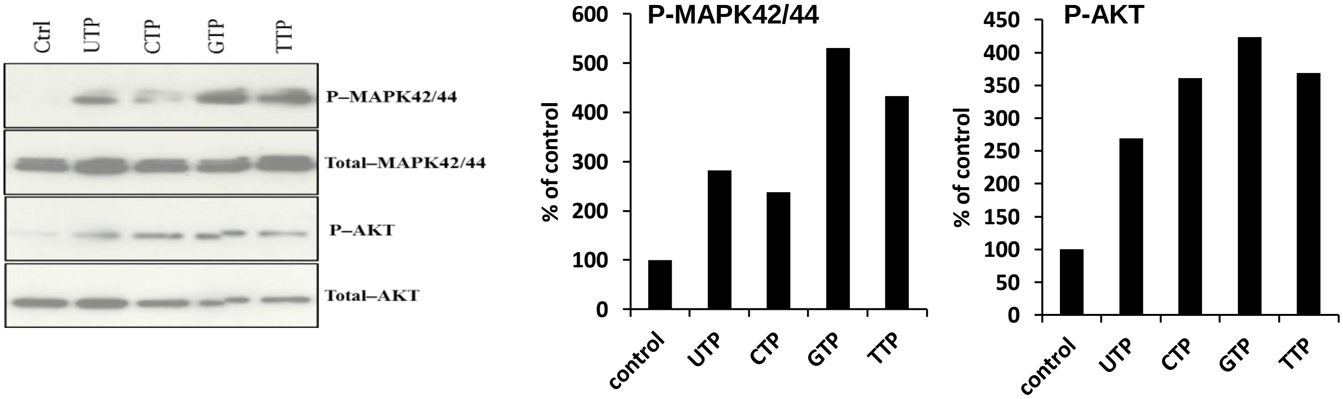

Supplementary Figure 4. **Extracellular TTP, UTP, CTP, and GTP stimulate human lung cancer cells.** Phosphorylation of p42/44 MAPK and AKT in the human NSCLC cell line HTB177 (**Panel A**) or the SCLC cell line CRL5853 (**Panel B**) stimulated for 5 min by the indicated nucleotides. The experiment was repeated twice, with similar results, and representative western blots are shown.
