# Supplementary material for: Extracellular nucleotides as novel, underappreciated pro-metastatic factors that stimulate purinergic signaling in human lung cancer cells
Source: Mol Cancer. 2015 Nov 24;14:201. doi: 10.1186/s12943-015-0469-z (PMC4657356; doi:10.1186/s12943-015-0469-z)
Supplement: Additional file 6: Figure S5. — Autocrine release of ATP plays a role in migration of lung cancer cells to HGF. Chemotaxis results of HTB177 cell line to HGF (10 ng/ml) in the absence or presence of apyrase (50U/ml). (PDF 87 kb) [file 12943_2015_469_MOESM6_ESM.pdf]

Supplementary Figure 5

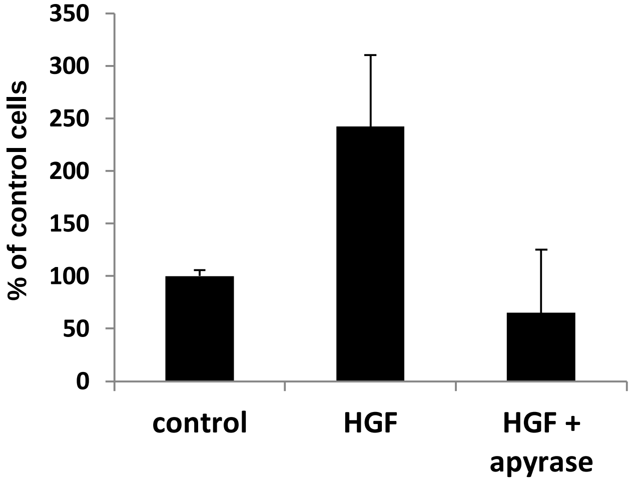

Supplementary Figure 5. Autocrine release of ATP plays a role in migration of lung cancer cells to HGF. Chemotaxis results of HTB177 cell line to HGF (10 ng/ml) in the absence or presence of apyrase (50U/ml).
